# The developing brain structural and functional connectome fingerprint

**DOI:** 10.1016/j.dcn.2022.101117

**Published:** 2022-05-20

**Authors:** Judit Ciarrusta, Daan Christiaens, Sean P. Fitzgibbon, Ralica Dimitrova, Jana Hutter, Emer Hughes, Eugene Duff, Anthony N. Price, Lucilio Cordero-Grande, J.-Donald Tournier, Daniel Rueckert, Joseph V. Hajnal, Tomoki Arichi, Grainne McAlonan, A. David Edwards, Dafnis Batalle

**Affiliations:** a Department of Forensic and Neurodevelopmental Science, Institute of Psychiatry, Psychology and Neuroscience, King’s College London, London, United Kingdom; b Centre for the Developing Brain, School of Imaging Sciences & Biomedical Engineering, King's College London, London, United Kingdom; c Center for Brain and Cognition (CBC), Universitat Pompeu Fabra, Barcelona, Spain; d Department of Electrical Engineering, ESAT/PSI, KU Leuven, Leuven, Belgium; e Wellcome Centre for Integrative Neuroimaging, FMRIB, Nuffield Department of Clinical Neurosciences, University of Oxford, UK; f Paediatric Neuroimaging Group, Department of Paediatrics, University of Oxford, UK; g Biomedical Image Technologies, ETSI Telecomunicación, Universidad Politécnica de Madrid & CIBER-BBN, Madrid, Spain; h Biomedical Image Analysis Group, Department of Computing, Imperial College London, London, United Kingdom; i Klinikum Rechts der Isar, Technical University of Munich, Munich, Germany; j Department of Bioengineering, Imperial College London, London SW7 2AZ, United Kingdom; k Children’s Neurosciences, Evelina London Children’s Hospital, Guy’s and St Thomas’ NHS Trust, London, United Kingdom; l MRC Centre for Neurodevelopmental Disorders, King’s College London, London, United Kingdom

**Keywords:** neonate, connectivity, brain networks, diffusion MRI, functional MRI, tractography, preterm

## Abstract

In the mature brain, structural and functional ‘fingerprints’ of brain connectivity can be used to identify the uniqueness of an individual. However, whether the characteristics that make a given brain distinguishable from others already exist at birth remains unknown. Here, we used neuroimaging data from the developing Human Connectome Project (dHCP) of preterm born neonates who were scanned twice during the perinatal period to assess the developing brain fingerprint. We found that 62% of the participants could be identified based on the congruence of the later structural connectome to the initial connectivity matrix derived from the earlier timepoint. In contrast, similarity between functional connectomes of the same subject at different time points was low. Only 10% of the participants showed greater self-similarity in comparison to self-to-other-similarity for the functional connectome. These results suggest that structural connectivity is more stable in early life and can represent a potential connectome fingerprint of the individual: a relatively stable structural connectome appears to support a changing functional connectome at a time when neonates must rapidly acquire new skills to adapt to their new environment.

## Highlights

• The brain structural connectome can be used to identify an individual at birth
• Functional connectivity is less stable and cannot be used as a fingerprint
• Age at first scan and days between scans impacts on similarity
• Structural and functional similarity appear closest in central sulcus
• Age at first scan significantly impacts on parietal functional similarity

## Graphical Abstract

**Figure:**
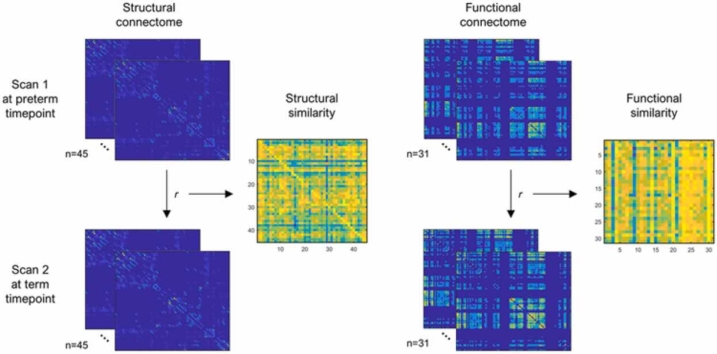

## 1 Introduction

Advances in neuroimaging technology have provided new means to investigate the human brain in vivo. This has enabled characterisation of connectomes delineating the structural and functional organisation of the brain at a macro-scale. These connectomes can be represented as large-scale matrices where each row and column correspond to brain subunit indices which may be structural or functional nodes, so that each matrix element describes ‘connectivity’ between two neural parts (Sporns, 2010, Sporns et al., 2005). The information contained in the functional and structural connectome of an individual is highly specific to that person and has been compared to a personal ‘fingerprint’ (Finn et al., 2015, Yeh et al., 2016). Although, the functional connectome has been demonstrated to be highly stable over multiple years after late adolescence (Horien et al., 2019), a delay in establishing a distinctive functional connectome through adolescence has been linked to mental health difficulties (Kaufmann et al., 2017). However, the structural and functional connectome of an infant differ from older age groups (Cao et al., 2017b), and the extent to which either is stable (i.e., reproducible at the level of the individual) is unknown. A better understanding of the extent of malleability of a given property of an individual’s brain and its relation to outcomes may guide personalised approaches to optimise child neurodevelopmental health.

The developing brain is governed by dynamic processes that transform an amalgam of a few cells in early gestation into a complex organ capable of rapidly processing and integrating information. The foetal period is marked by cortical migratory processes, with anomalies at this stage frequently leading to neuronal migration disorders and atypical brain structure (Kostović et al., 2014, Ten Donkelaar and Van der Vliet, 2004). A hallmark of the third foetal trimester is a change in the relative proportion of short and long range association fibres (Ouyang et al., 2019). These fast-changing microscopic developmental mechanisms quickly lead to reshaping of macrostructural features, which can be captured with Magnetic Resonance Imaging (MRI). For example, despite ongoing rapid growth, cortical folding features show remarkable high similarity between scans of the same subject across the first year following birth, suggesting a high self-similarity in cortical macrostructure between birth and the first 2 postnatal years (Duan et al., 2020). However, the stability of structural/functional network metrics and whether the connectome is ‘individual’ in early life has not been previously investigated. This is important to understand because, although the structural and functional connectome are highly inter-related and complimentary in adults (Sarwar et al., 2021), they represent models of brain connectivity with distinct developmental influences (for a review see Suárez et al., 2020).

Useful information about the perinatal brain and its subsequent maturation has already been acquired. In the perinatal brain, in parallel with structural changes, cortical neurons fire specific activity patterns which further regulate genetic expression and refine structure into functional systems (Kirkby et al., 2013, Tau and Peterson, 2010). Highly connected structural and functional nodes/hubs are crucial for information flow and further evolve as myelination matures (Morgan et al., 2018). A striking difference between the neonatal and the adult brain is that functional hubs, highly interconnected regions, are more restricted to somatosensory, auditory, visual and motor regions, unlike the higher order networks seen in adults (Cao et al., 2017a). In childhood, the identification accuracy of the functional connectome at rest has been estimated to be at 43% (Vanderwal et al., 2021), relative to 92% reported in adults (Finn et al., 2015), suggesting acquiring functional diversity and consequent uniqueness is part of the trajectory towards adulthood. However, the structural connectome fingerprint has only been investigated in adults and been shown to be relatively plastic globally but very stable within specific white matter bundles, such as the corpus callosum (Yeh et al., 2016). On the contrary, the neonatal brain is still immature and differs from the adult one, so it is possible that nature and extent of identifiable features of individuals’ structural and functional connectomes that are stable over time also differ.

Here, we investigate whether a structural and/or functional fingerprint is already established perinatally, by assessing the similarity of the structural and functional connectome of preterm born infants, who were scanned soon after birth and then again at term equivalent age. Unlike adults, the maturing brain is highly dynamic and undergoing rapid reorganisation. Therefore, we hypothesised that connectome similarity would be lowest when the time between scans was longest. In addition, brain activity becomes experience-driven during this early postnatal period and experiences increase daily as the infant interacts with the world ex-utero (Greenough et al., 1987, Khazipov and Luhmann, 2006). Thus, we also hypothesised that this constantly changing functional activity leads to experience-dependent changes in the functional connectome upon a (relatively) stable structural connectome.

## 2 Methods

### 2.1 Subjects

Research participants were prospectively recruited as part of the developing Human Connectome Project (dHCP), an observational, cross-sectional Open Science programme approved by the UK National Research Ethics Authority (14/LO/1169). Written consent was obtained from all participating families prior to imaging.

As part of the dHCP, a total of 63 subjects were scanned twice and had both functional and diffusion MRI data acquired. After pre-processing, 18 diffusion datasets were discarded due to poor registration to template space, resulting in a total of 45 subjects (26 males) with good quality diffusion MRI data. All infants were born preterm at a median of 32.29 weeks gestational age (GA) [range: 25.57–37], with their first scan acquired at a median of 35 weeks post menstrual age (PMA) [range: 29.29–37.43] and the second acquired at term equivalent age at a median of 41 weeks PMA [range: 38.43–44.86]. From the functional data, 18 subjects were discarded due to poor registration to template space and additional 14 had to be discarded due to significant signal loss during acquisition. Thus, there was a total of 31 subjects (21 males) with good quality functional connectome data, born at a median of 34.57 weeks GA [range: 27.57–37.00], had their first scan at a median of 35.57 weeks PMA [range: 30.86–37.43] and had their second scan at a median of 40.57 weeks PMA [range: 38.86–44.57]. The final sub-group with both good quality structural and functional data consisted of 26 subjects (16 males), born at a median age of 34.14 weeks [range: 28.71–37.00], with their first scan acquired at a median of 35.43 weeks PMA [range: 31.43–37.43] and the second acquired at a median of 40.93 weeks PMA [range: 38.86–44.86] (details described in Table 1).

**Table 1:** Descriptive sample characteristics (median [range]).

Group	GA at birth [*weeks*]	PMA - scan 1 [*weeks*]	PMA - scan 2 [*weeks*]
Structural, n=45 (26 males)	32.29 [25.57–37.00]	35.00 [29.29–37.43]	41.00 [38.43–44.86]

Functional, n=31 (21 males)	34.57 [27.57–37.00]	35.57 [30.86–37.43]	40.57 [38.86–44.57]

Structural and functional, n=26 (16 males)	34.14 [28.71–37.00]	35.43 [31.43–37.43]	40.93 [38.86–44.86]

### 2.2 Data acquisition

All subjects underwent Magnetic Resonance Imaging (MRI) scanning at the Evelina Newborn Imaging Centre, St Thomas’ Hospital, London, UK. Structural, diffusion and functional data was acquired using a 3 Tesla Philips Achieva system (Philips Medical Systems, Best, The Netherlands) with customised neonatal imaging system including a 32-channel phased-array head coil (Rapid Biomedical, Rimpar, Germany) (Hughes et al., 2017). Infants were studied during natural sleep following feeding and immobilisation in a vacuum evacuated bag (Med-Vac, CFI Medical Solutions, Fenton, MI, USA). Hearing protection (moulded dental putty in the external auditory meatus (President Putty, Coltene Whaledent, Mahwah, NJ, USA) and earmuffs (MiniMuffs, Natus Medical Inc., San Carlos, CA, USA)) and physiological monitoring (oxygen saturations, heart rate, axillary temperature) were applied before data acquisition. MR-compatible foam shielding was used for further acoustic noise attenuation. All scans were supervised by a neonatal nurse and/or paediatrician who monitored heart rate, oxygen saturation and temperature throughout the scan.

A total of 300 volumes of diffusion MRI were acquired over 19 min and 20 s with b-values of 400 s/mm^2^, 1000 s/mm^2^ and 2600 s/mm^2^ spherically distributed in 64, 88 and 128 directions respectively, with 20 b = 0 s/mm^2^ images and parameters: Multiband factor 4, SENSE factor 1.2, partial Fourier 0.86, acquired in-plane resolution 1.5 × 1.5 mm, 3 mm slices with 1.5 mm overlap, repetition time (TR)/ echo time (TE) of 3800/90 ms and 4 phase-encoding directions (Hutter et al., 2018). After reconstruction, image resolution was 1.5 mm isotropic. High-temporal-resolution BOLD fMRI optimised for neonates was acquired over 15 min 3 s (2300 volumes) using a multislice gradient-echo echo planar imaging (EPI) sequence with multiband excitation (multiband factor 9), TR 392 ms, TE 38 ms, flip angle 34°, and acquired spatial resolution 2.15 mm isotropic (Price et al., 2015). For clinical interpretation and registration purposes, a Turbo spin echo sequence (parameters: TR = 12 s, TE = 156 ms, SENSE factor 2.11 (axial) and 2.54 (sagittal)) was used to acquire high-resolution T2-weighted (T2w) images. The T2w axial and sagittal volumes originally acquired at 0.8 × 0.8 mm, 1.6 mm slices with 0.8 mm overlap were motion corrected and super-resolved to a final resolution of 0.8 mm isotropic (Cordero-Grande et al., 2018).

### 2.3 Image pre-processing and connectome construction

A neonatal specific segmentation pipeline (Makropoulos et al., 2014) was used to obtain tissue segmentation of each subject’s T2w images in native space. A neonatal adaptation (Shi et al., 2011) of the AAL atlas (Tzourio-Mazoyer et al., 2002) aligned to the dHCP high-resolution neonatal template (Schuh et al., 2018) was used to parcellate each subject's brain into 90 cortical and subcortical regions. Previously calculated tissue segmentation and T2w images were used as input for a non-linear registration based on a diffeomorphic symmetric image normalisation method (SyN) available in ANTS software (Avants et al., 2011) to bring the 90 regions neonatal atlas into the subject’s native space (Supplementary Table 1).

Pre-processing of diffusion MRI data and structural connectome construction was performed as previously reported (Taoudi-Benchekroun et al., 2022). Briefly, after hybrid SENSE reconstruction (Zhu et al., 2016), diffusion signal was denoised (Cordero-Grande et al., 2019), and susceptibility distortions were corrected (Andersson et al., 2003). A spherical harmonics and radial decomposition (SHARD) slice-to-volume reconstruction was applied to further correct motion effects and other artefacts (Christiaens et al., 2021). The N4 algorithm (Tustison et al., 2010) implemented in MRtrix (Tournier et al., 2019) was applied for bias field correction. Multi-tissue CSD (Jeurissen et al., 2014) using restricted anisotropic diffusion for brain tissue and free diffusion for fluid like features (Pietsch et al., 2019) was used to estimate fibre orientation distribution (FOD) in each brain voxel. Response functions for each tissue type were generated as the average from the response functions in an independent sub-group of 20 healthy term control neonates from the dHCP (Taoudi-Benchekroun et al., 2022). Multi-tissue log-domain intensity normalisation (Raffelt et al., 2017) was applied to FODs, and normalised brain tissue like FODs were used to generate 10 million streamlines with anatomically constrained probabilistic tractography (Smith et al., 2012) with biologically accurate weights (SIFT2) (Smith et al., 2015). The fibre density SIFT2 proportionality coefficient (μ) for each subject was obtained to achieve inter-subject connection density normalisation. Atlas parcellation and tissue maps in T2w native space were registered to diffusion space with a rigid registration using b = 0 volumes as target (Schnabel et al., 2001). The structural connectome of each infant was constructed in native diffusion space, by calculating the μ × SIFT2-weighted sum of streamlines connecting each pair of regions into a weighted adjacency matrix of size 90 × 90.

For functional data, all 2300 volumes of fMRI data acquired per participant were utilised without undergoing any scrubbing. Mean subject-wise framewise displacement (FD) was calculated to characterise motion in each time point (Supplementary Fig. 3A). In addition, we examined the relationship between global self-similarity and motion to take into consideration the potential effect of motion on the functional fingerprint (Supplementary Fig. 3B–D). Data were pre-processed using the Developing Human Connectome Project pipeline optimised for neonatal fMRI, detailed in (Fitzgibbon et al., 2020). In brief, susceptibility dynamic distortion together with intra- and inter-volume motion effects were corrected in each subject using a bespoke pipeline including slice-to-volume and rigid-body registration (Andersson et al., 2017). In order to regress out signal artefacts related to head motion, cardiorespiratory fluctuations and multiband acquisition, 24 extended rigid-body motion parameters were regressed together with single-subject ICA noise bespoke components identified with the FSL FIX tool (Oxford Centre for Functional Magnetic Resonance Imaging of the Brain’s Software Library, version 5.0) (Salimi-Khorshidi et al., 2014). Atlas parcellation and tissue maps were propagated from T2w native space using a boundary-based registration (Greve and Fischl, 2009). Average timeseries of each of the previously parcelled ROIs intersecting with GM regions were calculated in native fMRI space. Functional connectivity (FC) was calculated as the Pearson’s correlation of the signal between each pair of ROIs resulting in a matrix of size 90 × 90. Pearson’s correlation was selected as FC metric for consistency with previous fingerprinting literature (Finn et al., 2015, Vanderwal et al., 2021). In order to account for potential effects due to using different FC metrics in FC identifiability, additional functional connectivity matrices were also constructed using partial correlation between each pair of ROIs, controlling for the signal in the rest of the ROIs. Partial correlation was chosen as additional metric because it has been characterised as the best performing FC measure compared to Pearson’s correlations, which appear highly correlated with motion (Mahadevan et al., 2020). Negative correlations were not considered and set to zero.

### 2.4 Similarity analysis

#### 2.4.1 Global similarity – identifiability rate quantification

To identify the optimal network density threshold for structural and functional connectome similarity, we tested the percentage of correctly identified subjects for all possible thresholds (Supplementary Fig. 1). Based on this analysis, highest similarity value was obtained at many threshold values between 18% and 34%, which consistently identified the same set of subjects. Given there were no differences on similarity, we applied a 25% network density threshold to the structural and functional connectomes at time-point 1 for each subject. This connectivity matrix was then binarized and used as a mask for the connectivity matrix in time-point 2 ensuring that the same inter-regional connections were compared between the two time points. Spearman’s correlation between each matrix at time-point 1 and time-point 2 was calculated, resulting in a similarity matrix of 45 × 45 subjects for structural connectivity and 31 × 31 subjects for functional connectivity. If the self-similarity between time-point 1 and time-point 2 (diagonal correlation) was higher than the self-to-other-similarity, this was quantified as a successful match (Finn et al., 2015).

**Fig. 1:**
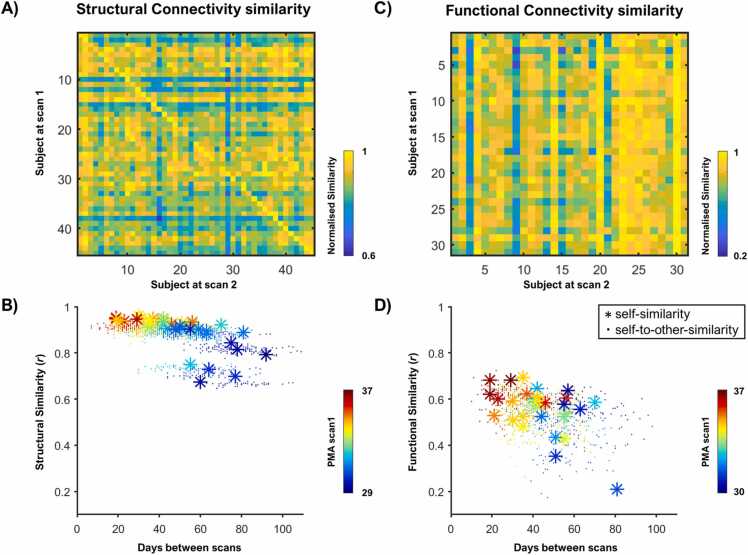
*Structural and Functional global similarity.* The correlation between the connectome of each subject at time-point 1 and 2 normalised by the maximum similarity to time-point 2 (each column) is depicted in the similarity matrix for structural connectivity (A) and functional connectivity (C). The correlations are then plotted against days between scans with a colour gradient showing the age of the subject at time-point 1 for structural data (B) and functional data (D). The stars represent the correlation between the connectome of a subject at time-point 1 with the connectome of the same subject at time-point 2 (i.e., self-similarity), and the dots represent the correlation of a subject at time-point 1 with a different subject at time-point 2 (i.e., self-to-other-similarity).

For visualisation purposes, all similarity values were normalised by scaling relative to the maximum correlation between time-point 1 and all other subjects at time-point 2 dividing by the maximum value in each row (i.e., for each row, value of 1 indicated the maximum match between time-point 1 and time-point 2). As shown in Fig. 1 this scaling results in a value of 1 in the diagonal when self-similarity is higher than any self-to-other-similarity value. If the value of 1 is not in the diagonal it indicates self-to-other-similarity is higher than self-similarity.

The same analysis was repeated for a sub-group of participants that had both structural and functional connectome data. This resulted in two 26 × 26 matrices containing structural and functional data, characterising the identifiability rate for the two modalities in the same individuals. The diagonal values of these matrices were extracted to provide median and range of structural and functional self-similarity.

#### 2.4.2 Age effect on self-similarity and self-to-other-similarity

In order to assess the effect of age in the structural and functional fingerprint, we first calculated the partial correlation between PMA at scan at time-point 1 and self-similarity (controlling for days between scans), and then the partial correlation between days between scans and self-similarity (controlling for PMA at time-point 1) for the whole group in each modality. Then, for the sub-group we converted the self-similarity and the self-to-other-similarity into z-scores for better visualisation of the effect of age on self-similarity. If the self-similarity z-score of a subject was higher than any of the self-to-other-similarity z-score, this would be equivalent to successfully matching a subject between time-point 1 and time-point 2, as in previous fingerprinting studies (Finn et al., 2015).

To further characterise the effect of age on self-similarity, we ran a general linear model analysis with PMA at time-point 1 and days between scans as independent variables and self-similarity as dependent variable. This allowed quantification of the beta coefficients of the age effect on self-similarity. To characterise self-to-other-similarity association with age difference between time-point 1 and time-point 2, we performed a linear mixed-effects model (LME) with days between scans as fixed effect and with subject at time-point1 dependent random effect for the intercept to account for repeated measures.

#### 2.4.3 Regional analysis

Given the distinct trajectories of maturation of subcortical and cortical regions, we repeated all the analysis described above in 7 clusters that represent larger anatomical areas: central, frontal, limbic, occipital, parietal, deep grey matter and temporal. These regions were composed of 8, 22, 14, 14, 10, 8, and 14 nodes respectively. The central cluster was composed of bilateral precentral and postcentral gyrus, paracentral lobules and supplementary motor areas. The frontal cluster was composed of superior, middle, inferior and orbitofrontal cortices, as well as the olfactory lobule and the rectus gyrus. The limbic cluster was composed of the insula, anterior and posterior cingulate, hippocampus and amygdala. The occipital cluster was composed of the calcarine, cuneus, lingual cortices together with superior, inferior and middle occipital gyrus and the fusiform. The parietal cluster was composed of the superior and inferior parietal gyrus, supramarginal, angular and precuneus gyrus. The deep grey matter cluster was composed of all basal ganglia structures and thalami. The temporal cluster was composed of the Rolandic operculum, the Heschl gyrus, and superior, middle and interior temporal cortices as well as the temporal poles. Supplementary Table 1 contains all 90 regions of the atlas and to which cluster they belong to. This allowed to calculate similarity identifiability rates, similarity z-scores and age linear models for each regional cluster.

## 3 Results

### 3.1 Whole-brain similarity

The structural connectome comparison between the scans at preterm and term equivalent ages yielded strong correlations not only among the scans of the same subject but also between the scans of different subjects (Fig. 1AB). The mean self-similarity was *r* = 0.90, ranging from *r* = 0.67 to *r* = 0.96, and the mean self-to-other-similarity was *r* = 0.88, ranging from *r* = 0.65 to *r* = 0.94. The identifiability rate was 28/45 (62.22%), representing the percentage of times self-similarity was higher than the self-to-other-similarity. Furthermore, we found a significant correlation between PMA at first scan and self-similarity values (i.e., the older a subject was at the first scan, the more “self-similar” their structural connectome was between scans), controlling for days between scans (*r* = 0.49, *p* = 0.006).

The functional connectome comparison showed less consistent results (Fig. 1CD). The correlation values between scans were lower compared to structural connectome similarities. The mean self-similarity was *r* = 0.56, ranging from *r* = 0.21 to *r* = 0.70, and the mean self-to-other-similarity was *r* = 0.54, ranging from *r* = 0.17 to *r* = 0.73. Thus, the identifiability rate for functional connectome similarity was 3/31 (9.68%). We observed no significant correlation between PMA at first scan and functional self- similarity, but there was a significant negative correlation between functional self-similarity and days between scans (*r* = − 0.39, *p* = 0.03). The same similarity rate was observed when testing partial correlation FC, 3/31(9.68%). However, mean self-similarity was just r = 0.12, ranging from r = 0.04 to r = 0.19, and mean self-to-other-similarity was r = 0.11, ranging from r = 0.02 to r = 0.22 (Supplementary Fig. 2 C–D). Similarity rate was lower for both Pearson (2/31(6.45%)) and partial correlation (1/31(3.24%)) when no thresholding or masking was applied (Supplementary Fig. 2 A–B and E–F).

**Fig. 2:**
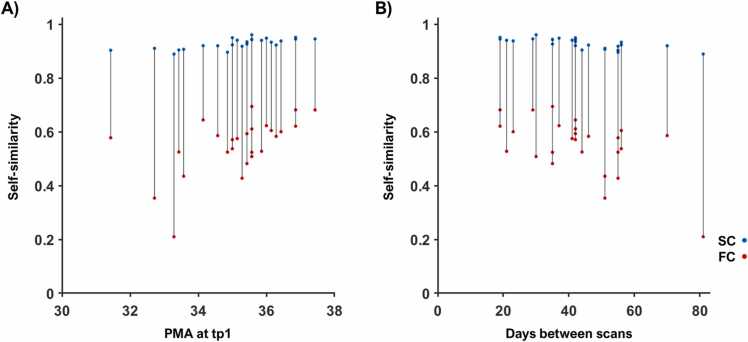
*Self-similarity for structural and functional connectivity.* The similarity correlation between the structural connectivity (SC) matrices between scans (blue) and between the functional connectivity (FC) matrices (red) is plotted against age at first scan (A) and against days between scans (B).

Closer inspection of the self-similarity for the structural and functional connectivity in a sub-group of participants with data available in both modalities confirmed that all subjects had higher similarity between their structural connectomes than their functional connectomes. The median structural self-similarity was *r* = 0.93, ranging from *r* = 0.89 to *r* = 0.96, and the median functional self-similarity was *r* = 0.55, ranging from *r* = 0.21 to *r* = 0.69. The identifiability rate for structural data was 18/26 (69.23%), in contrast with 3/26 (11.54%) for functional data (Fig. 2). As the structural and functional data examined were from the same individuals, age and time between scans was exactly matched.

**Fig. 3:**
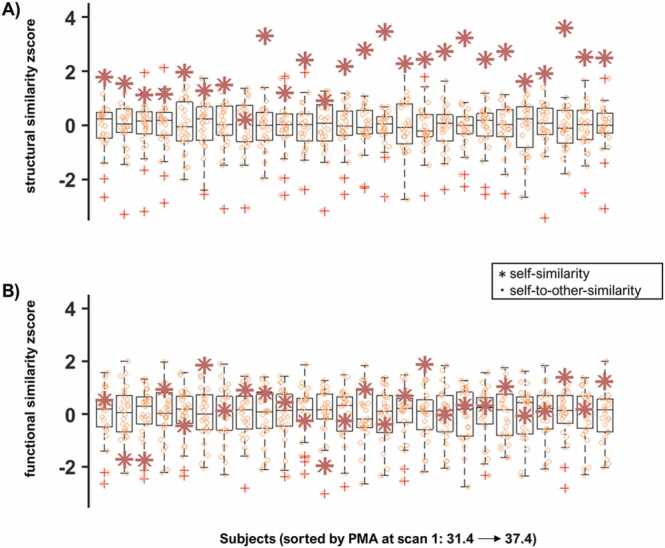
*Self-similarity and self-to-other-similarity z-scores arranged by PMA*. The boxplots show the similarity scores between time-point 1 and time-point 2 converted to z-scores for each participant arranged from left to right (youngest to oldest at time-point 1). The stars represent self-similarity and the circles represent self-to-other-similarity. The upper row depicts structural connectome similarity (A) and the bottom row shows functional connectome similarity (B).

Finally, we converted the self-similarity and self-to-other-similarity values into z-scores for each subject and sorted them based on age at time-point 1 to better visualise whether older subjects have a more identifiable whole-brain structural connectome (Fig. 3). Results show that the structural connectome was more stable than the functional connectome against variations on age or time between scans.

### 3.2 Age effect on sub-group similarity

The general linear model analysis for the sub-group with both structural and functional data further showed that age at time-point 1 has a significant effect on global structural connectome self-similarity (β = 0.006, p = 0.008). We observed no significant effect of days between scans on structural connectome self-similarity; and no effect of age at time point 1 or days between scans on global functional connectome self-similarity (Table 2).

**Table 2:** Beta coefficients and p-values for the effect of age at scan and days between scans on self-similarity.

	Structural Connectivity (SC)				Functional Connectivity (FC)			
	PMA at scan (time-point 1)		Days between scans		PMA at scan (time-point 1)		Days between scans	
	β	p	β	p	β	p	β	p
global	0.00611	0.01	-0.00058	0.01	0.02413	0.15	-0.00265	0.08
central	0.00126	0.72	-0.00024	0.44	-0.03211	0.06	-0.00258	0.09
frontal	0.00031	0.91	-0.00044	0.07	0.02662	0.31	-0.00270	0.25
limbic	0.00444	0.28	-0.00069	0.07	0.01875	0.28	-0.00187	0.23
occipital	-0.00107	0.74	-0.00046	0.13	0.06104	0.01	-0.00007	0.97
parietal	-0.00002	1.00	0.00029	0.60	0.08789	<0.01	0.00174	0.47
sub-cortical	-0.00150	0.61	-0.00019	0.47	-0.00186	0.91	-0.00069	0.64
temporal	0.00216	0.44	-0.00015	0.56	0.01117	0.54	-0.00041	0.80

The LME analysis showed age at first scan also has a significant effect on global structural connectome self-to-others-similarity (β = 0.006, p < 0.001), but there was no significant effect of days between scans. Unlike what was observed for self-similarity, both days between scans (β = − 0.001, p = 0.003) and age at time-point 1 (β = 0.023, p < 0.001) had a significant effect on global functional connectome self-to-others-similarity.

### 3.3 Regional similarity

Regional comparison of structural connectomes showed lower identifiability rate compared to the global metrics. The limbic cluster (insula, cingulate, hippocampus and amygdala) showed highest identifiability rate at 11/45 (24.44%), followed by frontal regions at 10/45 (22.22%), occipital at 7/45 (15.56%), central at 5/45 (11.11%), both parietal and deep GM at 4/45 (8.89%) and temporal at 3/45 (6.67%).

**Fig. 4:**
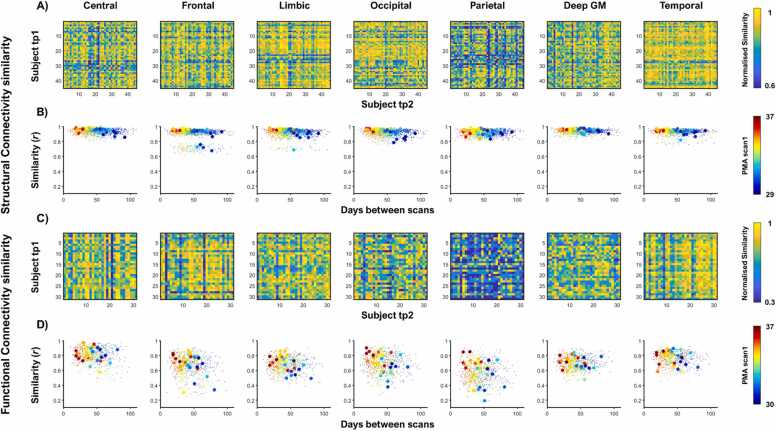
*Structural and Functional cluster-wise similarity.* Normalised similarity matrices together with plots to depict the association of the similarity correlation with days between scans underneath are shown for structural connectivity (A, B) and functional connectivity (C, D). These figures are presented in different columns for different anatomical clusters: somatosensory-motor or central region, frontal, limbic, occipital, parietal, deep grey matter, and temporal.

Functional connectome comparisons yielded similar identifiability rates compared to whole-brain metrics. They were still qualitatively lower than structural similarity identifiability rates, except for the parietal cluster. Central and parietal regions showed the highest identifiability rate of 3/31 (9.68%). Limbic regions showed an identifiability rate of 2/31 (6.45%) and frontal, occipital, sub-cortical and temporal clusters showed an identifiability rate of 1/31 (3.23%) (Fig. 4).

**Fig. 5:**
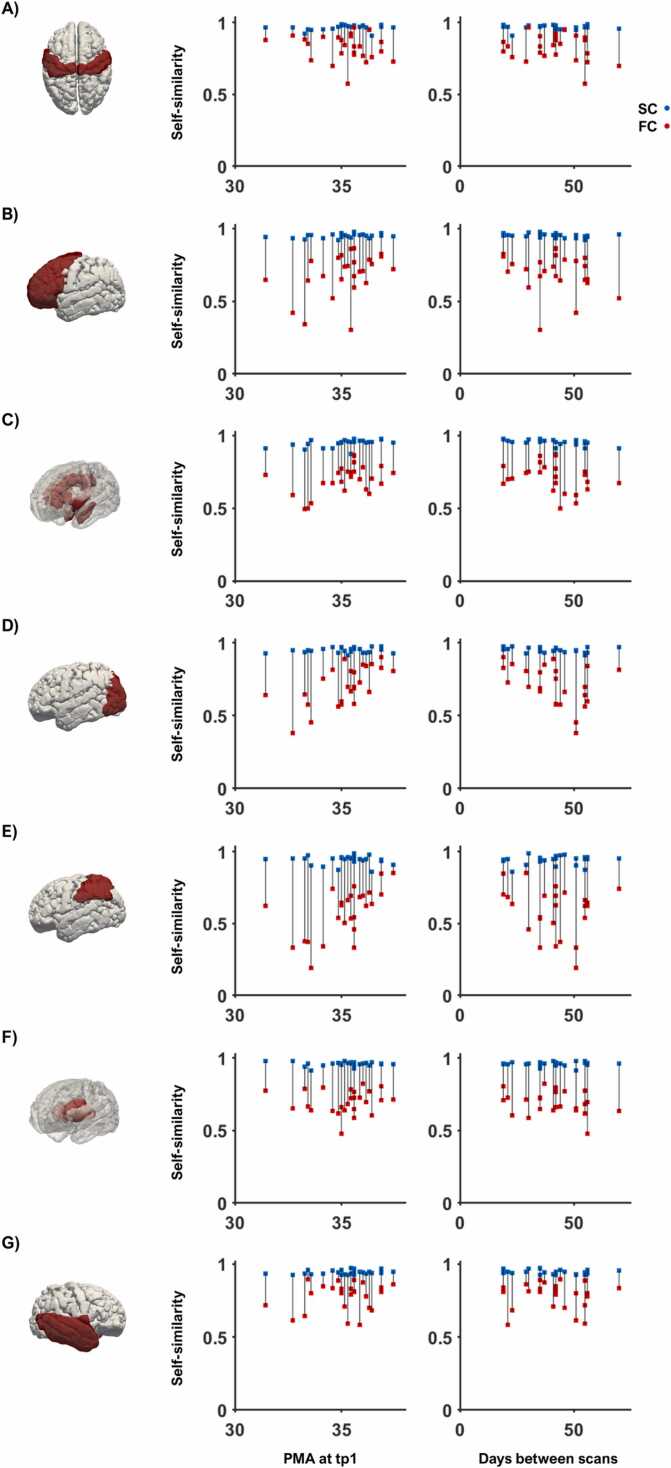
*Regional self-similarity for structural and functional connectivity cluster-wise.* The structural self-similarity (blue) and the functional self-similarity (red) is plotted against age at first scan (PMA at time-point 1) in the first column and against days between scans in the second column for central (A), frontal (B), limbic (C), occipital (D), parietal (E), deep grey matter (F) and temporal (G) cluster. Grey lines provide visual guidance to match structural and functional similarity values of the same subject.

Closer qualitative inspection of the self-similarity for the structural and functional connectivity in the sub-group of participants with both structural and functional connectomes showed structural and functional self-similarity closer together in the central cluster and more dispersed in the frontal cluster (Fig. 5). However, functional similarity identifiability values were always lower than structural identifiability rates in this sub-group.

### 3.4 Age effect on regional self-similarity

The generalised linear model run independently for each cluster in the sub-group with both structural and functional data showed that age at time-point 1 has a significant effect on functional connectome similarity in the parietal region after Bonferroni correction (β = 0.088, p = 0.003). We observed no significant effect of age or days between scans on any other cluster for functional nor structural self-similarity after multiple comparisons correction (Table 2).

## 4 Discussion

In the current study we used a unique set of longitudinal high quality structural and functional neonatal brain MRI data from the Developing Human Connectome Project to investigate the status of the connectome fingerprint at an early stage of neurodevelopment. To do so, we selected data from preterm born infants that were scanned soon after birth and then again at term equivalent age. Our results show that the whole-brain structural connectome can already identify an older individual at birth. In contrast, the whole-brain functional connectome changes more noticeably between scan timepoints, so individual identification is less stable based on their functional connectome regardless of their age at birth or time between scans.

### 4.1 A structural connectivity fingerprint is present during the perinatal period

During the perinatal period, the brain undergoes marked micro and macrostructural changes (Batalle et al., 2017, Kunz et al., 2014, Pietsch et al., 2019). Nevertheless, our observations suggest that by the normal time of birth, an individual’s brain structural connectome is relatively stable. This suggests that the individual template of structural connectivity is predominately genetically determined, in the absence of an external insult. Consistent with this, macroscale structural white matter tractography has been shown to be highly heritable with axial diffusivity, radial diffusivity and fractional anisotropy of commissural fibres found to have the highest genetic influence and association fibres the least (Lee et al., 2015). By term equivalent age, the neonatal brain has an established framework of thalamocortical fibres, a large abundance of u-shaped cortico-cortical fibres, and visible long range association pathways such as the cingulum bundle and callosal fibres (Takahashi et al., 2012). The abundance of u-shaped cortico-cortical fibres at term is in line with an early establishment of cortical folding patterns that remain individually unique throughout the first two postnatal years (Duan et al., 2020). Thus, the acquisition of a stable structural connectome appears to coincide with the attainment of more mature brain structural appearance and may therefore be a marker of maturity. This fits with the observed relationship between higher structural connectome self-similarity and an older age at the time of the first scan. Alongside self-similarity, we also observed high self-to-other-similarity values in the structural connectome which likely represents the development of common macroscale features (structural connectivity backbone).

### 4.2 Functional connectivity fingerprints are immature in preterm born infants

Task-based fMRI studies in neonates have demonstrated that the primary sensory cortices (e.g. somatosensory, auditory, olfactory and visual) are capable of processing external stimuli and are undergoing activity-dependent maturation during the perinatal period (Adam-darque et al., 2018, Allievi et al., 2016, Anderson et al., 2001, Arichi et al., 2010, Baldoli et al., 2015, Dall’Orso et al., 2018, Lee et al., 2012, Perani et al., 2010). Resting state studies have reported significant age-dependent increases in functional short-range connectivity in the somatosensory, visual, auditory and language networks throughout the perinatal time window, further suggesting rapid functional plasticity and maturation within these systems (Cao et al., 2017a). These changes in local degree centrality may represent functional reshaping of primary resting state networks that resemble adult networks by term equivalent age, while higher-order association networks appear immature (Eyre et al., 2021). Such fast reorganisation across multiple functional systems during the perinatal time window may explain why we observed such few cases where self-similarity was higher than self-to-others-similarity.

Our results markedly contrast with those of previously reported work in children. To our knowledge, the youngest previously investigated population for connectome similarity analysis to date has been a sample of 6-year-old children, where they reported an identification rate of 43% between resting state functional connectomes (Vanderwal et al., 2021). Another study in children aged 7–15 years reported high correlation coefficients between functional connectivity matrices of the same participant, almost on par with adult similarity values (Horien et al., 2019). A potential argument for these differences could be the metric used, given that using partial correlations for FC in adults have yielded higher identifiability rates in comparison to Pearson’s correlation or dynamic connectivity (Menon and Krishnamurthy, 2019). However, we observed no differences in similarity rates between partial correlations and Pearson’s correlation. While our results indicate that a functional connectome fingerprint is barely present in preterm born infants in the perinatal period, future studies should investigate whether identifiability differs in term born infants and when this starts to increase between infancy and childhood.

An additional factor that should be considered is the impact of motion artefacts on functional connectivity (Mahadevan et al., 2020, Parkes et al., 2018). Adult studies have reported low identifiability based on motion parameters relative to functional connectivity self-similarity, suggesting motion is not contributing to high identifiability (Finn et al., 2015). However, we observed a negative correlation of mean FD with global functional self-similarity (Supplementary Fig. 3C–D). This correlation was absent when we selected a subgroup of subjects with mean FD lower than 0.3 at both timepoints (Supplementary Fig. 4B–D). These observations suggest motion may be an additional factor contributing to low functional fingerprinting capacity observed in our sample. The functional connectome might thus appear more stable in a sample with low motion estimates. Nevertheless, we have shown functional connectivity self-similarity is consistently lower than structural connectivity self-similarity in this population (Fig. 2), even for subjects with low mean FD motion estimates (Supplementary Fig. 4E–F) supporting our hypothesis that functional fingerprint is immature in preterm babies.

### 4.3 Impact of age at first scan and days between scans on similarity

To disentangle the impact of inter-scan interval and age at first scan on similarity of the structural and functional connectome, we examined a sub-group of 26 neonates who had both data types. We observed a significant effect of age at time-point 1 on the global structural self-similarity. This effect might be determined by the individual-specific developmental trajectory of white matter microstructure and not necessarily indicative of an adult-like unique structural connectome (Ouyang et al., 2019).

The absence of any significant effect of age on global functional self-similarity at this early stage of development might be explained by the reshaping of long-range functional connectivity, which matures within the first postnatal year (Damaraju et al., 2014). However, we cannot exclude the possibility that the low functional connectome self-similarity in the perinatal period is a false negative. For example, there may be a non-linear effect that cannot be captured with a linear model or an anatomical parcellation might not be optimal to characterise the stability of the functional connectome in early development. Future studies focusing in functional connectome similarity should investigate if an appropriate parcellation derived from functional data or multi-modal information based parcellations (Glasser et al., 2016) yields higher self-similarity rates.

Interestingly, we observed the same age effect on global structural connectome self-to-other-similarity. This suggests certain age dependent organisation patterns are also common across subjects. The same was observed for functional self-to-other-similarity, which significantly decreased with longer intervals between scans being compared and increased with older age at time point 1. The low self-similarity, combined with strong self-similarity-to others and its age dependency roots for time constrained functional changes at a developmental time where there is a strong mixture of finely tuned spontaneous neural activity patterns and sensory experience dependent mechanisms (Hanganu-Opatz, 2010, Toyoizumi et al., 2013). The strong dependency of age and similarity patterns across subjects may also be related to genetic expression patterns that are highly dynamic and fast changing during this time of development (Silbereis et al., 2015).

### 4.4 Regional fingerprinting of the connectome

Brain development broadly follows a posterior to anterior maturational trajectory (Huttenlocher and Dabholkar, 1997) with sensory systems developing before higher order networks (Cao et al., 2017b). Structural connectome identifiability rate was lower when investigating regions separately, suggesting global metrics with more data points are more informative as a fingerprint when using structural data. Later developing networks such as frontal and limbic cortices had the highest structural self-similarity. Given the late maturation of these regions, we speculate the high self-similarity might be driven by the fact that these regions are undergoing the least amount of structural change during the interval between scans.

We saw the highest identifiability rate in the central cluster of the functional connectome. This suggests that functional sensory-motor networks can provide higher identifiability rates in babies, while in comparison frontal-parietal structures appear more unique in adults (Finn et al., 2015). However, it will require a larger sample of data specifically comparing somatosensory and frontal regions in infants and adults to test this hypothesis. In addition, structural and functional coupling is a sign of maturation which appears more robust after adolescence (Baum et al., 2020, Sarwar et al., 2021). Thus, the closer resemblance between structure and function self-similarity in the central cluster might be indicative of the relatively mature state of somatosensory and motor cortices in the perinatal time window.

### 4.5 Limitations

The main limitation of this study is that to assess the connectome fingerprint across weeks in the perinatal period ex-utero there is no other option but to investigate a preterm born population. Hence, to what extent the fingerprint is affected by preterm birth or is representative of normal development will have to be investigated in older infant cohorts or using foetal MRI. Another limitation pertains the multiple developmental factors which may influence the acquired signal in different ways and consequently the connectome. Despite the robustness of SIFT to characterise structural connections (Smith et al., 2015), using the most advanced pipelines available for neonatal fMRI data processing (Fitzgibbon et al., 2020), and being very stringent on data quality measures, the potential influence of developmental factors on the signal is out of our control. For diffusion data, developmental changes such as cortical folding or tissue water content reduction among others can affect the signal differently at different ages. Similarly, developmental effects which may influence the BOLD signal in different ways might relate to vascular density or neurovascular coupling, which can affect the sensitivity and specificity at different ages. Therefore, MR signal changes on the similarity values reported might be beyond differences in the neural “fingerprint”. On this line, it is also important to note we used anatomical parcellations to characterise the functional nodes and future studies should investigate whether diverse functional parcellation methods mimic or differ from the findings reported in this study. In addition, we suggest future studies should further investigate how to disentangle potential effects of motion when investigating the immature functional connectome, given that motion effects are likely to differ from effects reported with adults.

## 5 Conclusion

The brain structural connectome fingerprint is already present in the perinatal period: it is relatively stable and individually unique at this stage of development. The identification features of functional connectivity are more complex to interpret, potentially being too dynamic or immature to provide a fingerprint. Region-wise analysis suggested that the functional fingerprint in early development might be more stable within regional clusters, although identifiability rates were still higher for structural data. Future studies should investigate regional differences throughout development, the association of the global structural fingerprint to developmental outcome, and whether genetic or environmental risk impact the stability of the fingerprint.

## Data availability

The dHCP is an open-access project. The imaging and collateral data can be downloaded by registering at https://data.developingconnectome.org/.

Derived data including structural and functional connectivity networks used in this study are available in github.com/code-neuro/neonatalconnectomefingerprint/.

## Funding

This work was supported by the European Research Council under the European Union Seventh Framework Programme (FP/2007–2013)/10.13039/100010663ERC Grant Agreement no. 319456. The authors acknowledge infrastructure support from the National Institute for Health Research (NIHR) Mental Health Biomedical Research Centre (10.13039/100014461BRC) at South London, Maudsley NHS Foundation Trust, 10.13039/501100000764King's College London and the NIHR-BRC at Guys and St Thomas’ Hospitals NHS Foundation Trust (GSTFT). The authors also acknowledge support in part from the Wellcome Engineering and Physical Sciences Research Council (10.13039/501100000266EPSRC) Centre for Medical Engineering at King’s College London [WT 203148/Z/16/Z] and the 10.13039/501100000265Medical Research Council (UK) [MR/K006355/1]. Additional sources of support included the Sackler Institute for Translational Neurodevelopment at King’s College London, the European Autism Interventions (EU-AIMS) trial and the EU AIMS-2-TRIALS, a European Innovative Medicines Initiative Joint Undertaking under Grant agreements nos. 115300 and 777394, the resources of which are composed of financial contributions from the European Union’s Seventh Framework Programme (Grant FP7/2007–2013). DC is supported by the 10.13039/501100003130Flemish Research Foundation (FWO, fellowship [12ZV420N]). TA is supported by a MRC Clinician Scientist Fellowship [MR/P008712/1]. DE received support from the Medical Research Council Centre for Neurodevelopmental Disorders, King’s College London [MR/N026063/1]. DB received support from a Wellcome Trust Seed Award in Science [217316/Z/19/Z]. The views expressed are those of the author(s) and not necessarily those of the NHS, the NIHR or the Department of Health. The funders had no role in the design and conduct of the study; collection, management, analysis, and interpretation of the data; preparation, review, or approval of the manuscript; and decision to submit the manuscript for publication.

## Declaration of Competing Interest

The authors declare that they have no known competing financial interests or personal relationships that could have appeared to influence the work reported in this paper.
