# Supplementary material for: The developing brain structural and functional connectome fingerprint
Source: Dev Cogn Neurosci. 2022 May 20;55:101117. doi: 10.1016/j.dcn.2022.101117 (PMC9344310; doi:10.1016/j.dcn.2022.101117)
Supplement: Supplementary file 1 — Supplementary material [file mmc1.pdf]

The developing brain structural and functional connectome fingerprint: Supplementary Material

Judit Ciarrusta[a,b], Daan Christiaens[b,c], Sean P. Fitzgibbon[d], Ralica Dimitrova[a,b], Jana Hutter[b], Emer Hughes[b], Eugene Duff[d,e], Anthony N Price[b], Lucilio Cordero-Grande[b,f], J-Donald Tournier[b], Daniel Rueckert[g,h], Joseph V Hajnal[b], Tomoki Arichi[b,i,j], Grainne McAlonan[a,k,*], A David Edwards[b,k,*], Dafnis Batalle [a,b,*†]

a. Department of Forensic and Neurodevelopmental Science, Institute of Psychiatry, Psychology and Neuroscience, King's College London, London, United Kingdom
b. Centre for the Developing Brain, School of Imaging Sciences & Biomedical Engineering, King's College London, London, United Kingdom
c. Department of Electrical Engineering, ESAT/PSI, KU Leuven, Leuven, Belgium
d. Wellcome Centre for Integrative Neuroimaging, FMRIB, Nuffield Department of Clinical Neurosciences, University of Oxford, UK
e. Paediatric Neuroimaging Group, Department of Paediatrics, University of Oxford, UK
f. Biomedical Image Technologies, ETSI Telecomunicación, Universidad Politécnica de Madrid & CIBER-BBN, Madrid, Spain
g. Biomedical Image Analysis Group, Department of Computing, Imperial College London, London, United Kingdom
h. Klinikum Rechts der Isar, Technical University of Munich, Munich, Germany
i. Department of Bioengineering, Imperial College London, London, SW7 2AZ, United Kingdom
j. Children's Neurosciences, Evelina London Children's Hospital, Guy's and St Thomas' NHS Trust, London, United Kingdom
k. MRC Centre for Neurodevelopmental Disorders, King's College London, London, United Kingdom

Supplementary Figure 1

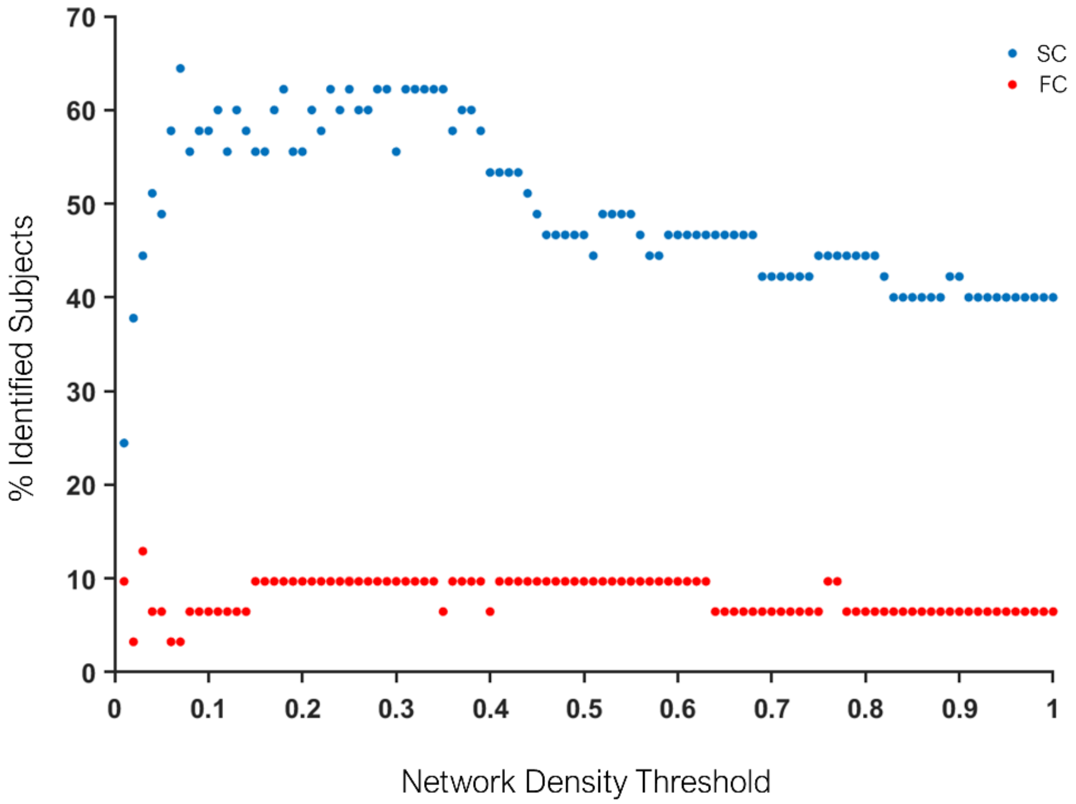

Supplementary Figure 1. Network Density dependent identifiability rate for structural (blue -SC) and functional (red-FC) connectivity.

Supplementary Figure 2

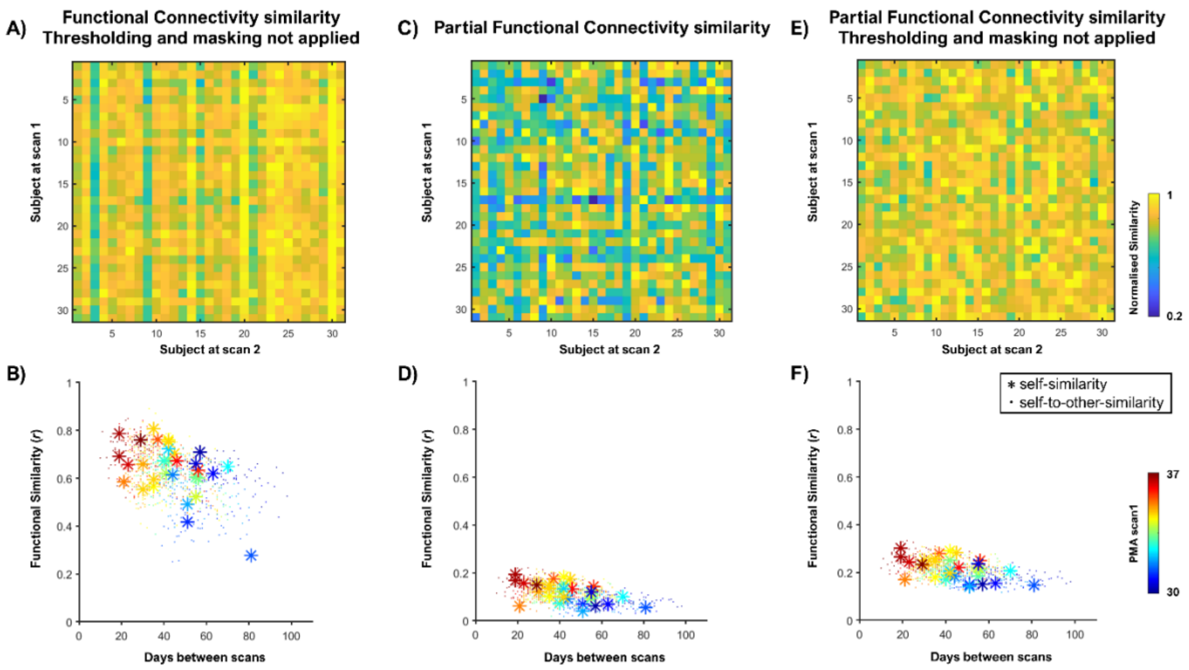

Supplementary Figure 2. Functional global similarity for different functional connectivity metrics. The correlation between the connectome of each subject at time-point 1 and 2 normalised by the maximum similarity to tp2 (each column) is depicted in the similarity matrix for functional connectivity using Pearson's correlation without any network density threshold or masking (A), using partial correlation with a threshold of 25% network density (C) and using partial correlation without any network density threshold or masking (E).`The correlations are then plotted against days between scans with a colour gradient showing the age of the subject at time-point 1 for full functional connectivity (B), for threshold partial functional connectivity (D) and un-threshold partial functional connectivity (F). The stars represent the correlation between the connectome of a subject at time-point 1 with the connectome of the same subject at time-point 2 (i.e., self-similarity), and the dots represent the correlation of a subject at time-point 1 with a different subject at time-point 2 (i.e., self-to-other-similarity).

Supplementary Figure 3

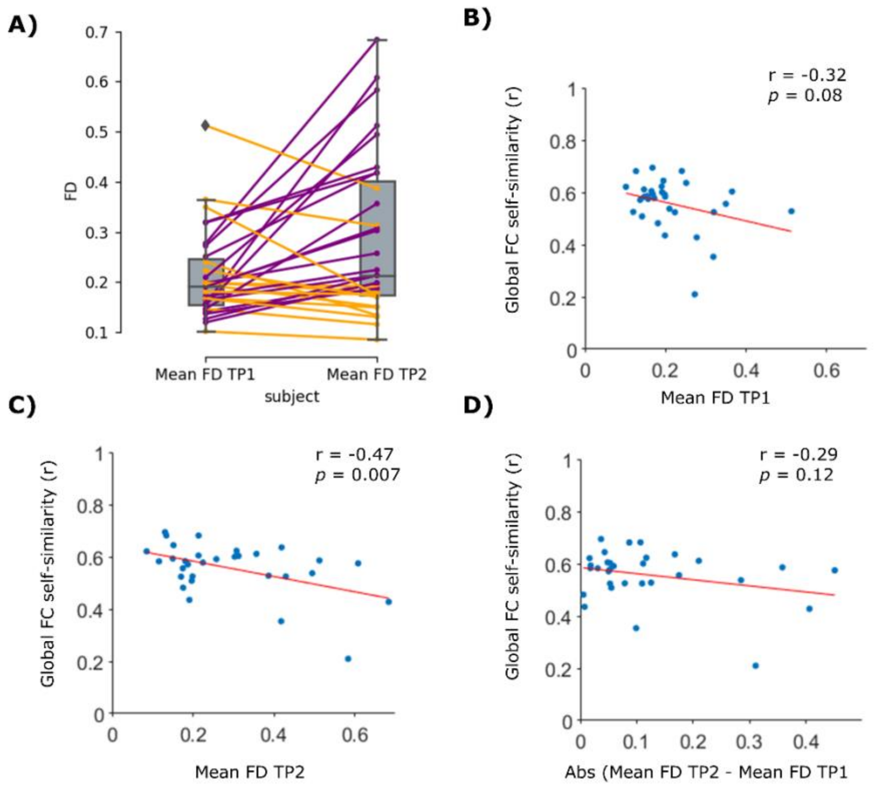

Supplementary Figure 3. Mean Framewise Displacement (FD) effect on global functional self-similarity. Mean FD per participant at time-point 1 (TP1) and time-point 2 (TP2) are shown in a boxplot, infants that had higher mean FD at TP2 relative to TP1 appear in purple, while infants that had lower mean FD appear in orange (A). To characterize the impact of motion, global functional self-similarity is plotted against mean FD at TP1 (B), mean FD at TP2 (C), and the absolute difference in mean FD between time-points (D). A linear regression fit line is shown in red in every plot (B-D).

Supplementary Figure 4

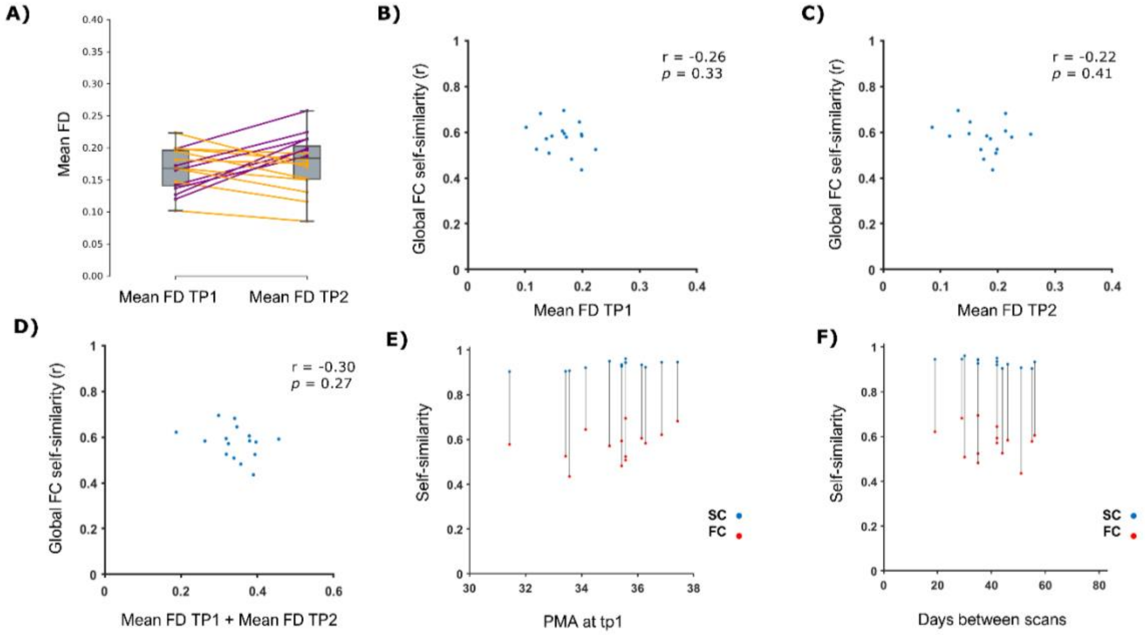

Supplementary Figure 4. Low mean Framewise Displacement (FD) effect on global functional self-similarity. Mean FD per participant with a cut-off mean FD of 0.3 at time-point 1 (TP1) and time-point 2 (TP2) are shown in a boxplot, infants that had higher mean FD at TP2 relative to TP1 appear in purple, while infants that had lower mean FD at TP2 appear in orange (A). To characterize the impact of motion, global functional self-similarity is plotted against mean FD at TP1 (B), mean FD at TP2 (C), and the cumulative motion (sum of mean FD at TP1 and TP2) (D). A linear regression fit line is shown in red in every plot; Pearson's r and uncorrected p-value are also reported (B-D). Among the 16 subjects with low motion values, the 14 that had both structural and functional data are shown in E-F. The self-similarity correlation between the structural connectivity (SC) matrices between scans (blue) and between the functional connectivity (FC) matrices (red) is plotted against age at first scan (E) and against days between scans (F).

Supplementary Table 1. Connectome nodes, anatomical regions, and clusters

Node	Region	Cluster	Node	Region	Cluster
1	Precentral gyrus left	Central	46	Cuneus right	Occipital
2	Precentral gyrus right	Central	47	Lingual gyrus left	Occipital
3	Superior frontal gyrus (dorsal) left	Frontal	48	Lingual gyrus right	Occipital
4	Superior frontal gyrus (dorsal) right	Frontal	49	Superior occipital gyrus left	Occipital
5	Orbitofrontal cortex (superior) left	Frontal	50	Superior occipital gyrus right	Occipital
6	Orbitofrontal cortex (superior) right	Frontal	51	Middle occipital gyrus left	Occipital
7	Middle frontal gyrus left	Frontal	52	Middle occipital gyrus right	Occipital
8	Middle frontal gyrus right	Frontal	53	Inferior occipital gyrus left	Occipital
9	Orbitofrontal cortex (middle) left	Frontal	54	Inferior occipital gyrus right	Occipital
10	Orbitofrontal cortex (middle) right	Frontal	55	Fusiform gyrus left	Occipital
11	Inferior frontal gyrus (opercular) left	Frontal	56	Fusiform gyrus right	Occipital
12	Inferior frontal gyrus (opercular) right	Frontal	57	Postcentral gyrus left	Central
13	Inferior frontal gyrus (triangular) left	Frontal	58	Postcentral gyrus right	Central
14	Inferior frontal gyrus (triangular) right	Frontal	59	Superior parietal gyrus left	Parietal
15	Orbitofrontal cortex (inferior) left	Frontal	60	Superior parietal gyrus right	Parietal
16	Orbitofrontal cortex (inferior) right	Frontal	61	Inferior parietal lobule left	Parietal
17	Rolandic operculum left	Temporal	62	Inferior parietal lobule right	Parietal
18	Rolandic operculum right	Temporal	63	Supramarginal gyrus left	Parietal
19	Supplementary motor area left	Central	64	Supramarginal gyrus right	Parietal
20	Supplementary motor area right	Central	65	Angular gyrus left	Parietal
21	Olfactory left	Frontal	66	Angular gyrus right	Parietal
22	Olfactory right	Frontal	67	Precuneus left	Parietal
23	Superior frontal gyrus (medial) left	Frontal	68	Precuneus right	Parietal
24	Superior frontal gyrus (medial) right	Frontal	69	Paracentral lobule left	Central
25	Orbitofrontal cortex (medial) left	Frontal	70	Paracentral lobule right	Central
26	Orbitofrontal cortex (medial) right	Frontal	71	Caudate left	Deep Grey Matter
27	Rectus gyrus left	Frontal	72	Caudate right	Deep Grey Matter
28	Rectus gyrus right	Frontal	73	Putamen left	Deep Grey Matter
29	Insula left	Limbic	74	Putamen right	Deep Grey Matter

30	Insula right	Limbic	75	Pallidum left	Deep Grey Matter
31	Anterior cingulate gyrus left	Limbic	76	Pallidum right	Deep Grey Matter
32	Anterior cingulate gyrus right	Limbic	77	Thalamus left	Deep Grey Matter
33	Middle cingulate gyrus left	Limbic	78	Thalamus right	Deep Grey Matter
34	Middle cingulate gyrus right	Limbic	79	Heschl gyrus left	Temporal
35	Posterior cingulate gyrus left	Limbic	80	Heschl gyrus right	Temporal
36	Posterior cingulate gyrus right	Limbic	81	Superior temporal gyrus left	Temporal
37	Hippocampus left	Limbic	82	Superior temporal gyrus right	Temporal
38	Hippocampus right	Limbic	83	Temporal pole (superior) left	Temporal
39	ParaHippocampal gyrus left	Limbic	84	Temporal pole (superior) right	Temporal
40	ParaHippocampal gyrus right	Limbic	85	Middle temporal gyrus left	Temporal
41	Amygdala left	Limbic	86	Middle temporal gyrus right	Temporal
42	Amygdala right	Limbic	87	Temporal pole (middle) left	Temporal
43	Calcarine cortex left	Occipital	88	Temporal pole (middle) right	Temporal
44	Calcarine cortex right	Occipital	89	Inferior temporal gyrus left	Temporal
45	Cuneus left	Occipital	90	Inferior temporal gyrus right	Temporal
